# Decreased Renal Function is Associated with Heart Failure Readmissions

**DOI:** 10.7759/cureus.3122

**Published:** 2018-08-09

**Authors:** Mohinder R Vindhyal, Sinan Khayyat, Adnan Shaaban, Brent A Duran, K. James Kallail

**Affiliations:** 1 Internal Medicine, University of Kansas School of Medicine - Wichita, Wichita, USA; 2 Internal Medicine, Lebanese University, Beirut, LBN

**Keywords:** heart failure, renal failure

## Abstract

Introduction

Heart failure (HF) is one of the most common causes of hospitalization and readmissions. Approximately six million Americans are living with HF. Among patients with HF, hospitalization rate in the United States is higher for those over age 65, making it one of the leading causes of hospitalization in this age group. Furthermore, about 15% of those who were hospitalized with HF were readmitted within 30 days and 30% within 60 days. HF and chronic kidney disease (CKD) share many risk factors; therefore, it is expected that CKD is more prevalent in HF. About 50% of patients with HF also have concomitant CKD. Those patients have been found to have an increased risk of mortality and morbidity. This risk increases as glomerular filtration rate (GFR) decreases. Strategies to reduce the hospitalization rate in patients with HF include optimizing evidence-based drug and device therapies, addressing the causes of HF, treating comorbidities, and improving management of care. In our study, we aim to find an association between HF and the patient’s renal function as well as the GFR level. This study investigates the effect of renal function on HF morbidity and readmission rate.

Methods

We performed a retrospective study looking at 132 patients who were admitted to the hospital with HF and compared their measured GFR at three key time periods: admissions, discharges, and readmissions at 30 days. A Pearson product-moment correlation coefficient was calculated to determine the association between the GFR and readmission in HF admission cases.

Results

There is a statistically significant difference in the readmission rate based on the change in GFR between admission and discharge (Admit GFR – Discharge GFR; *t* = 2.28; p < 0.05). We found that patients who were readmitted in 30 days had an average decrease in GFR by 2.46 ml/min/1.73 m^2^, whereas patients with a lower readmission rate had an average increase in GFR by 1.92 ml/min/1.73 m^2^.

Conclusion

A decline in renal function due to hospitalization in patients with renal failure is associated with an increase in readmission for HF. Providers should be cognizant of the need to optimize renal function as well as cardiac function during hospitalization.

## Introduction

Heart failure (HF) is a major public health issue, and it is associated with a high rate of mortality, morbidity, and hospital admission [1]. HF in the United States costs the healthcare system approximately $39 billion annually. It has an estimated prevalence of 5.8 million cases in the United States alone. Of these cases, 80% are ≥65 years [1-3]. It is also responsible for 300,000 deaths per year [3]. Furthermore, about 15% of those who were hospitalized with HF were readmitted within 30 days and 30% within 60 days [4]. About 50% of patients with HF also have concomitant chronic kidney disease (CKD). Those patients have been found to have an increased risk of mortality and morbidity. This risk increases as glomerular filtration rate (GFR) decreases [5]. According to the Framingham Heart Study, HF is associated with a 30-day mortality of 10%, one-year mortality of 20%-30%, and a five-year mortality of 45%-60% despite the advances in treatment and management [6-8]. Acute Decompensated Heart Failure National Registry (ADHERE) study reported that patients with HF have coronary artery disease, hypertension, diabetes, and kidney disease in 57%, 73%, 44%, and 60% of the cases, respectively [9-10]. HF and CKD share many risk factors; therefore, it is expected that CKD is more prevalent in HF patients. The HF patients with concurrent CKD have been found to have an increased mortality and morbidity, and this risk increases as GFR decreases [11]. There is no debate about the importance of the association between heart function and kidney function and how this plays a major role in HF prognosis. This interaction is bidirectional as heart disease can affect renal function and renal disease can affect cardiac function. As an example, the reduced renal function is associated with an increased mortality in patients with HF [11]. In addition, cardiovascular disease is responsible for up to 50% of deaths in patients with renal failure [11]. Management of CKD plays an important role in HF prognosis. Angiotensin converting enzyme (ACE) inhibitors and angiotensin receptor blockers lead to a reduction in mortality in patients with systolic HF [12]. These medications are underutilized in patients with HF for many reasons. For example, the increase in serum creatinine soon after the initiation of these drugs leads to the perception that this increase is a result of a decline in kidney function despite its actual reversibility [13-14]. In addition, ACE inhibitors and angiotensin receptor blockers reduce kidney disease progression; therefore, both are cardio-renal protective in HF patients with CKD. This will be a valuable consideration because of the increased mortality associated with CKD in HF [15]. Most of the HF patients have at least one or more concurrent comorbidities. It is estimated that around 25% of the patients who are hospitalized for HF are readmitted within 30 days and 30% within 60–90 days [16-17]. A reduced GFR is associated with an increased mortality risk in patients with HF whether it presents at baseline or it develops during therapy for HF. The prevalence of moderate to severe reductions in GFR (less than 60 ml/min/1.73 m2) in patients with HF has ranged from 30% to 60% in large clinical studies [18-19]. The magnitude of this effect can be illustrated by the findings in a systematic review of 16 studies with over 80,000 patients [18]. Khan et al. showed the prognostic effect of renal dysfunction in patients with HF. Patients with more than 10 ml/min/year fall in GFR have a significant increase in mortality [20]. This observation is clinically important because baseline GFR is a predictor of mortality in both acute and chronic HF. It is estimated that mortality will increase by approximately 7% for every 10 ml/min reduction in GFR [18, 20]. Our study investigates the association between HF and CKD and the effect of worsening renal function during hospitalization on HF prognosis and 30 days readmission rate in relation to GFR stratification.

## Materials and methods

Research and study design

This is a retrospective cohort study.

Participants

Some 132 patients with HF were evaluated over two years (from Jan 1, 2010 to Dec 31, 2012) from a Kansas Hospital database. The patients were older than 18 years of age and less than 89 years of age. There was no risk to the patients.

Instruments

Patients meeting all inclusion criteria had the following parts of their charts reviewed: history and physical, lab data, admission echocardiogram, and discharge documents. The study variables included: medical record number, age, race, type of admitting physician, type of admission, primary and secondary diagnosis, blood pressure, heart rate, respiratory rate, weight, height, body mass index, vitals on discharge, length of stay, time of rehospitalization – primary and secondary diagnosis, surgical history, lab data including sodium, potassium, blood sugar, blood urea nitrogen, hemoglobin, hematocrit, mean corpuscular volume, magnesium, brain natriuretic peptide, uric acid level, erythrocyte sedimentation rate, troponin, phosphate level, albumin level, urinalysis, and creatinine. Cardiovascular data such as electrocardiogram, echocardiogram showing ejection fraction, heart catheterizations were also recorded. The study defined HF as either any diastolic HF or a systolic ejection fraction less than 40% as reported on an echocardiogram performed during the current hospital admission. GFR was collected to assess the renal function, and the lowest creatinine value during the current hospitalization was selected for use as the best estimate of the patient’s baseline value. Data on race were collected to determine the estimated GFR using the CKD epidemiology collaboration (CKD-EPI) equation [12].

Procedures/measurements

This study was approved by the institutional review boards at the University of Kansas School of Medicine-Wichita and Via Christi Hospitals of Wichita, Kansas. A convenience sample of 132 subjects meeting the study criteria was assigned by the study director for review. The charts were reviewed by investigators, and the aforementioned data were collected and entered into a data sheet stored on password-secured computers. The data sheets were compiled into one data sheet for analysis. A convenience sample was used because this was intended to be a pilot study to obtain data and trends regarding the association.

## Results

**Figure 1:**
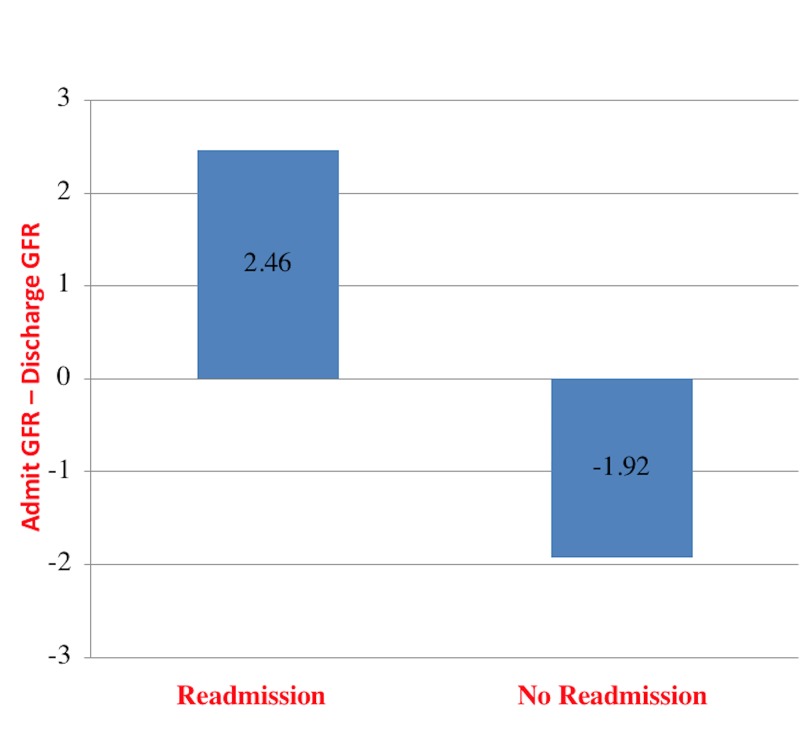
Average change in glomerular filtration rate (GFR) between admission and discharge by a 30-day readmission status.

A Pearson product-moment correlation coefficient was calculated to determine the association between the GFR and HF readmissions. Summary statistics were calculated for all the variables. All subjects met the eligibility criteria. A total of 132 subjects were included. Patients were stratified by their GFR into two main groups: one group with a GFR ≥ 60 ml/min/1.73 m^2^ and the other with a GFR < 60 ml/min/1.73 m^2^. The GFR of patients readmitted in 30 days had an average decrease by 2.46 ml/min/1.73 m^2^ whereas patients not readmitted had an average increase in GFR by 1.92 ml/min/1.73 m^2^ (Admit GFR – Discharge GFR; *t* = 2.28; p < 0.05) (Figure 1).

There is also a statistically significant difference in the 30-day readmission rate depending on the average brain natriuretic peptide (BNP) value on admission. The BNP values for the 30-day readmission versus no readmission in the GFR >/=60 group were 1297.8 and 1089.3 pg/ml (p < 0.01) while in the GFR <60 group the values were 2217.5 and 1088.2 pg/ml (p < 0.01) respectively. Another statistically significant difference was found in the readmission rate according to the average creatinine level at admission. The creatinine values for the 30-day readmission versus no readmission were 2.83 and 1.90 mg/dl (Table 1). There was no statistically significant difference in the readmission rate based on neither age, length of stay, hemoglobin level, left ventricle end systolic dimension, left ventricle ejection fraction nor patients on beta blockers, ACE inhibitors, angiotensin receptor blockers, or diuretics (Table 2).

**Table 1:** Average admission parameters by GFR and readmission status. GFR: glomerular filtration rate; BNP: brain natriuretic peptide.

Average values	GFR >/= 60 ml/min/1.73 m^2^	GFR < 60 (ml/min/1.73 m^2^)	p Value
*Average admit BNP (pg/ml)*			
30-Day readmission	1297.8	2217.5	p < 0.01
No readmission	1089.3	1088.2	
Average admit creatinine			
30-Day readmission	0.82	2.83	p < 0.01
No readmission	0.84	1.90	
Average age			
30-Day readmission	69.6	73	p > 0.05
No readmission	70	75.3	
Average length of stay (days)			
30-Day readmission	6.6	6.9	p > 0.05
No readmission	5.0	5.1	

**Table 2:** Differences by GFR and admission status. GFR: glomerular filtration rate; ACE: angiotensin converting enzyme; ARB: angiotensin II receptor blocker.

	Number of patients with GFR >/= 60 (ml/min/1.73 m^2^)	Number of patients with GFR < 60 (ml/min/1.73 m^2^)	p Value
Left ventricular end diastolic volume (cm)			
<5.8; 30-Day readmission	5	7	p > 0.05
<5.8; No readmission	15	29	
>5.8; 30-Day readmission	2	6	
<5.8; No readmission	4	9	
Left ventricular ejection fraction			
<50%; 30-Day readmission	12	5	p > 0.05
<50%; No readmission	16	37	
>50%; 30-Day readmission	8	4	
>50%; No readmission	10	28	
Hemoglobin (g/dl)			
>/=11; 30-Day readmission	12	5	p > 0.05
>/=11; No readmission	16	37	
<11; 30-Day readmission	8	4	
<11; No readmission	10	28	
ACE inhibitors or ARBs (yes)			
30-Day readmission	7	11	p > 0.05
No readmission	16	32	
B-Blockers (yes)			
30-Day readmission	7	16	p > 0.05
No readmission	19	50	
Potassium-sparing diuretics (yes)			
30-Day readmission	3	2	p > 0.05
No readmission	4	8	
Loop diuretics (yes)			
30-Day readmission	10	14	p > 0.05
No readmission	23	65	

## Discussion

Our study highlights the effect of renal dysfunction and its management on HF morbidity. It is not surprising that the management of CKD will lead to an improvement in the prognosis of HF. Also, conversely, improving the cardiac function leads to improved renal perfusion. This is due to the interactive relationship between the cardiac and renal functions as both have many common risk factors [11]. It is hypothesized that HF will lead to worsening of kidney function by two main mechanisms. First, HF patients will have a decrease in stroke volume and thus a lower cardiac output. This will lead to activation of the renin-angiotensin-aldosterone system (RAAS). All of these factors together will lead to a low renal perfusion and a decreased eGFR [21-22]. Another hypothesis suggests that high central venous congestion due to right ventricular dysfunction leads to a decline in eGFR [21]. Worsening renal function during HF hospitalizations leads to poor outcomes [11]. In addition, HF patients with worsening CKD have a significantly higher rate of readmission compared with those with preserved renal function during hospitalization [18-19]. Our study demonstrates that patients with an average decrease (Admit GFR - Discharge GFR) of 2.46 ml/min/1.73 m^2^ in GFR baseline level had a significantly higher 30-day readmission rate compared to patients who had an average increase in their GFR baseline level by 1.92 ml/min/1.73 m^2^ (Admit GFR - Discharge GFR). Therefore, it is important to optimize both cardiac and renal functions in patients with concomitant HF and CKD to improve the HF outcomes. Our study also supports prior reports highlighting the effects of worsening GFR during hospitalization on HF morbidity and mortality [10, 18, 20, 23]. We have also found an increased risk for hospitalization in HF patients depending on the average creatinine level during hospitalization. Patients with increased creatinine level had an increased 30-day readmission rate. Patients with preserved kidney function with a GFR less than 60 ml/min/1.73 m^2^ had an increased risk of hospitalization. In addition, patients with an average creatinine level of 2.83 mg/dl had an increased risk of hospitalization compared to those who had an average creatinine level of 1.90 mg/dl. Our findings are similar to prior reports that demonstrate significantly worse outcomes based on incremental increases in serum creatinine

Study limitations

Due to the small sample size, conducting it at one medical care center in the Midwest and over a limited period of time leads to limit the generalizability of the study on a larger population. The GFR calculation had been taken at three different time intervals and might be altered due to different variables. Thus, it is not necessarily reflecting the actual kidney function. However, it establishes a base for further research in evaluating the role of renal function on HF morbidity and mortality. Berkson’s bias is a concern in our study due to conducting it only in an inpatient setting. Conducting this study at more than one health care center with a larger representing population sample should help to lower the limitations on it and to eliminate the different modifiable variables’ effects and any possible confounding factors. Although the average renal function during hospitalization fluctuated minimally during hospitalization, these limited changes had a significant effect on the HF prognosis and the readmission rate. Thus, controlling the renal function, in HF patients with CKD or a preserved renal function, will lead to better outcomes and decreased mortality and morbidity. In addition, it is important to appropriately control the modifiable risk factors which affect the cardiac and renal disease.

## Conclusions

Several factors are associated with HF readmissions; further work is required to identify the modifiable components, which aid in continuing efforts to develop strategies that allow hospitals to achieve reductions in readmission rates. One such small factor is optimizing the renal function in HF patients admitted with HF exacerbation. Even though there are small gains in reducing the readmission rates, there is still much work to be done in achieving a true reduction in the rate of HF readmissions.

## References

[1] Writing Group Members, Mozzafarian D, Benjamin EJ (2016). Heart disease and stroke statistics—2016 update: a report from the American Heart Association. Circulation.

[2] Mosterd A, Hoes AW, de Bruyne MC (1999). Prevalence of heart failure and left ventricular dysfunction in the general population; The Rotterdam Study. Eur Heart J.

[3] Bui AL, Horwich TB, Fonarow GC (2011). Epidemiology and risk profile of heart failure. Nat Rev Cardiol.

[4] Gheorghiade M, Vaduganathan M, Fonarow GC, Bonow RO (2013). Rehospitalization for heart failure problems and perspectives. J Am Coll Cardiol.

[5] Ahmed A, Campbell RC (2008). Epidemiology of chronic kidney disease in heart failure. Heart Fail Clin.

[6] Go AS, Mozaffarian D, Roger VL (2013). Heart disease and stroke statistics—2013 update: a report from the American Heart Association. Circulation.

[7] Levy D, Kenchaiah S, Larson MG (2002). Long-term trends in the incidence of and survival with heart failure. N Engl J Med.

[8] Mozaffarian D, Benjamin EJ, Go AS (2015). Heart disease and stroke statistics-2015 update: a report from the American Heart Association. Circulation.

[9] Adams KF Jr, Fonarow GC, Emerman CL (2005). Characteristics and outcomes of patients hospitalized for heart failure in the United States: rationale, design, and preliminary observations from the first 100,000 cases in the Acute Decompensated Heart Failure National Registry (ADHERE). Am Heart J.

[10] Heywood JT, Fonarow GC, Costanzo MR (2007). High prevalence of renal dysfunction and its impact on outcome in 118,465 patients hospitalized with acute decompensated heart failure: a report from the ADHERE database. J Card Fail.

[11] Al-Ahmad A, Rand WM, Manjunath G (2001). Reduced kidney function and anemia as risk factors for mortality in patients with left ventricular dysfunction. J Am Coll Cardiol.

[12] Hunt SA, Abraham WT, Chin MH (2005). ACC/AHA 2005 guideline update for the diagnosis and management of chronic heart failure in the adult. A report of the American College of Cardiology/American Heart Association Task Force on practice guidelines (writing committee to update the 2001 guidelines for the evaluation and management of heart failure). Developed in collaboration with the American College of Chest Physicians and the International Society for Heart and Lung Transplantation: endorsed by the Heart Rhythm Society. Circulation.

[13] Masoudi FA, Rathore SS, Wang Y (2004). National patterns of use and effectiveness of angiotensin-converting enzyme inhibitors in older patients with heart failure and left ventricular systolic dysfunction. Circulation.

[14] Ahmed A, Allman RM, DeLong JF, Bodner EV, Howard G (2002). Age-related underutilization of angiotensin-converting enzyme inhibitors in older hospitalized heart failure patients. South Med J.

[15] Rothwell PM (2005). Subgroup analysis in randomised controlled trials: importance, indications, and intepretation. Lancet.

[16] Krumholz HM, Lin Z, Keenan PS (2013). Relationship between hospital readmission and mortality rates for patients hospitalzed with acute myocardial infarction, heart failure, or pneumonia. J Am Med Assoc.

[17] Fonarow GC, Stough WG, Abraham WT (2007). Characteristics, treatments, and outcomes of patients with preserved systolic function hospitalized for heart failure a report from the OPTIMIZE-HF registry. J Am Coll Cardiol.

[18] Smith GL, Lichtman JH, Bracken MB (2006). Renal impairment and outcomes in heart failure: systematic review and meta-analysis. J Am Coll Cardiol.

[19] Komukai K, Ogawa T, Yag Hi (2008). Decreased renal function as an independent predictor of re-hospitalization for congestive heart failure. Circ J.

[20] Khan NA, Ma I, Thompson CR (2006). Kidney function and mortality among patients with left ventricular systolic dysfunction. J Am Soc Nephrol.

[21] Mullens W, Abrahams Z, Francis GS (2009). Importance of venous congestion for worsening of renal function in advanced decompensated heart failure. J Am Coll Cardiol.

[22] Testani JM, McCauley BD, Kimmel SE, Shannon RP (2010). Characteristics of patients with improvement or worsening in renal function during treatment of acute decompensated heart failure. Am J Cardiol.

[23] Damman K, Navis G, Voors AA (2007). Worsening renal function and prognosis in heart failure: systematic review and meta-analysis. J Card Fail.

